# Adaptive dynamic range shift (ADRIFT) quantitative phase imaging

**DOI:** 10.1038/s41377-020-00435-z

**Published:** 2021-01-01

**Authors:** Keiichiro Toda, Miu Tamamitsu, Takuro Ideguchi

**Affiliations:** 1 Department of Physics, The University of Tokyo, Tokyo, 113-0033 Japan; 2 Institute for Photon Science and Technology, The University of Tokyo, Tokyo, 113-0033 Japan; 3 PRESTO, Japan Science and Technology Agency, Saitama, 332-0012 Japan

**Keywords:** Phase-contrast microscopy, Imaging and sensing

## Abstract

Quantitative phase imaging (QPI) with its high-contrast images of optical phase delay (OPD) maps is often used for label-free single-cell analysis. Contrary to other imaging methods, sensitivity improvement has not been intensively explored because conventional QPI is sensitive enough to observe the surface roughness of a substrate that restricts the minimum measurable OPD. However, emerging QPI techniques that utilize, for example, differential image analysis of consecutive temporal frames, such as mid-infrared photothermal QPI, mitigate the minimum OPD limit by decoupling the static OPD contribution and allow measurement of much smaller OPDs. Here, we propose and demonstrate supersensitive QPI with an expanded dynamic range. It is enabled by adaptive dynamic range shift through a combination of wavefront shaping and dark-field QPI techniques. As a proof-of-concept demonstration, we show dynamic range expansion (sensitivity improvement) of QPI by a factor of 6.6 and its utility in improving the sensitivity of mid-infrared photothermal QPI. This technique can also be applied for wide-field scattering imaging of dynamically changing nanoscale objects inside and outside a biological cell without losing global cellular morphological image information.

## Quantitative phase imaging: Sensitivity boost

A scheme that significantly enhances the sensitivity of quantitative phase imaging (QPI) could help benefit a range of applications in optical imaging and sensing. Keiichiro Toda and co-workers from The University of Tokyo, Japan call their scheme adaptive dynamic range shift quantitative phase imaging (ADRIFT-QPI). It works by “cancelling out” the sample-induced large optical phase delays (OPDs) by wavefront shaping and measuring the remaining small OPDs with a boosted sensitivity by dark-field microscopy. The team tested the approach with mid-infrared photothermal QPI measurements and confirmed a ~6.6 times sensitivity improvement. It is thought that the approach may also prove beneficial for detecting movement of nanoscale particles such as exosomes or viruses inside and outside a biological cell and slight OPD changes accompanied by the intracellular molecular dynamics.

## Introduction

Phase imaging^1–19^ provides morphological phase-contrast of transparent samples and is widely used in various fields, especially in biological science, because morphological features of micrometre-scale specimens provide valuable information on complex biological phenomena. Quantitative phase imaging (QPI)^1–6^ is the most powerful method for studying cellular morphology among various phase imaging methods, such as phase-contrast^7^ and differential-interference-contrast^8^ imaging, because it is able to accurately measure the optical phase delay (OPD) caused by a sample. This quantitative nature enables, for example, cellular dry mass and growth rate analyses^9^, which have been recognized as new tools for single-cell analysis. Since the minimum detectable OPD of conventional QPI (~10 mrad) is already small enough to clearly observe the surface roughness of a glass slide that limits the detectable sample-induced OPD, sensitivity improvement of QPI, especially of the temporal OPD sensitivity, has not been intensively explored. However, recent developments in pump-probe-type perturbative QPI techniques, such as mid-infrared (MIR) photothermal QPI^10–13^, have shown that temporal differential analysis of consecutively measured images can cancel out the substrate background and reveal small OPD changes. Thus, sensitivity improvement of QPI is highly demanded in this context. In addition, wide-field scattering imaging techniques using dark-field (DF)^14,15^ or interferometric scattering (iSCAT)^16,17^ have also used the concept of differential image analysis to observe dynamically changing small signals originating from fast-moving nanoscale scattering objects in a slowly moving microscale environment. They have been mostly used for investigating simple biomimicking systems^16,17^ and, more recently, applied to measure gold nanoparticles on cell membranes^18,19^. However, the limited dynamic range of DF imaging causes decreased sensitivity in the presence of large-OPD objects (>1 rad) such as cells. On the other hand, iSCAT is not a technique for quantitatively and comprehensively measuring a complex structure of specimens because it is only sensitive to the medium boundaries. These features prevent simultaneous quantitative detection of the global cellular structure and small scattering signals. To measure the dynamic motion of small particles, such as exosomes, liposomes and viruses, inside and outside a living cell, we need to detect small signals on top of the large background from the cell with a high measurement dynamic range in the manner of QPI.

**Fig. 1:**
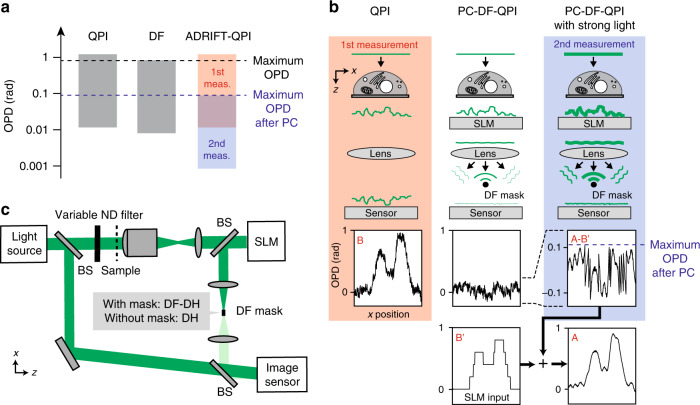
Principle of ADRIFT-QPI. **a** Dynamic range of phase imaging: (left) conventional QPI, (centre) dark-field imaging, (right) ADRIFT-QPI. We assume a standard CMOS image sensor where a full well capacity of ~10,000 e^−^/pixel is used. **b** Principle of dynamic range expansion in ADRIFT-QPI. The left column shows the first measurement, where the large-OPD distribution of the sample (B) is measured by QPI with plane wave illumination. The centre column shows the situation of PC-DF-QPI where the DF mask blocks the unscattered light by phase cancellation with the SLM. The right column shows the second measurement, which is PC-DF-QPI with strong light illumination, allowing for dynamic-range-shifted highly sensitive measurement. The dynamic-range-expanded OPD distribution of the sample (A) can be computationally reconstructed by adding the PC-DF-QPI measurement result (A–B′) to the SLM input OPD map (B′). **c** Optical implementation of ADRIFT-QPI. In this work, off-axis DH is used as a QPI technique. DH and DF-DH are switchable in a single setup by insertion/removal of the DF mask. The phase-only SLM is placed in the sample conjugate plane for wavefront shaping, while the DF mask is placed in the Fourier plane for spatially filtering the unscattered light. The illumination light intensity on the sample can be changed with a neutral density (ND) filter placed in front of the sample. BS: beamsplitter

Conventional QPI techniques, however, are not made to detect small OPDs because the image sensor is dominantly exposed to strong unscattered light (also known as zeroth-order diffracted light), which brings no information about the sample morphology, and the shot noise determines the dynamic range of the measurement. With a commonly used image sensor, the measurable OPD range is from ~10 mrad to ~1 rad in shot-noise-dominant imaging conditions, limiting the temporal sensitivity of the system^6^ (Fig. 1a). The sensitivity can be improved by increasing the photon flux of the scattered light reaching the image sensor because this light contains morphological information of the sample. This is realized by dark-field (DF) imaging^14,15^, where the undesired strong unscattered light is rejected with a spatial filter in the Fourier plane. Its dynamic range can be shifted to the smaller-OPD regime by increasing the illumination light to the level where the brightest spot caused by the largest OPD in the field of view (FOV) nearly saturates the image sensor. However, the sensitivity improvement cannot be significant when the sample has a global structure that causes a large OPD (~1 rad) because the dynamic range is “pinned” to this value (Fig. 1a). Therefore, the DF imaging technique is also not applicable to observing small OPD changes embedded in microscale large-OPD structures. For example, single-cell imaging falls into this situation in any case. To date, all existing phase imaging techniques, not only DF imaging, fail to simultaneously measure subtle OPD changes and large OPDs because the dynamic range is pinned to the larger side. To address this challenge, it is necessary to expand the dynamic range of the OPD measurement.

Here, we propose and demonstrate a method to expand the dynamic range of phase imaging, which we call adaptive dynamic range shift quantitative phase imaging (ADRIFT-QPI). It works by separately measuring the large- and small-OPD distributions of a sample and seamlessly connecting them (Fig. 1a). In addition to measuring large OPDs with the conventional QPI, we measure small OPDs with a new technique called phase-cancelling dark-field quantitative phase imaging (PC-DF-QPI), which is enabled by a wavefront shaping technique in the dark-field QPI (DF-QPI) configuration. Our proof-of-concept experiments demonstrate dynamic range expansion (sensitivity improvement) of QPI measurement by a factor of 6.6, which corresponds to a 44 times speed improvement, and show significant sensitivity improvement of MIR photothermal QPI. The concept of this technique promises remarkable advancement of label-free imaging with its high sensitivity.

## Results

### Principle of ADRIFT-QPI

The working principle of the dynamic range expansion is illustrated in Fig. 1b. In the first measurement, the large-OPD distribution is measured by conventional QPI with plane wave illumination. Then, the large-OPD distribution is optically cancelled by wavefront shaping with a phase-only spatial light modulator (SLM)^20–22^ such that the light reverts to a quasi-plane wave that can be focused around a single spot in the Fourier plane where a single dot spatial mask (DF mask) selectively rejects the focused light. The DF spatial mask allows only a small amount of light, which deviates from the plane wave, to reach the image sensor so that the illumination photon flux on the sample can be increased without saturating the sensor. Thus, in the second measurement performed by PC-DF-QPI with stronger illumination light, the dynamic range of the measurement is shifted to the smaller-OPD regime. The sensitivity of PC-DF-QPI determines that of ADRIFT-QPI. Note that to maintain the phase quantitativeness, we implement a technique to perform QPI in the DF configuration (DF-QPI). Finally, a dynamic-range-expanded OPD image of the sample is computationally reconstructed by adding the measured PC-DF-QPI image to the SLM input OPD map. To guarantee high sensitivity, it is necessary to precisely calibrate the response of the SLM with respect to the input numerical value so that the SLM phase ambiguity becomes lower than that offered by the sensitivity of PC-DF-QPI. Note that the OPD image obtained in the first measurement cannot be used for the computational reconstruction due to the digitization noise of the SLM (i.e., B ≠ B′ in Fig. 1b).

### Optical layout of ADRIFT-DH

The optical implementation of the system is presented in Fig. 1c. In this work, we implement off-axis digital holography (DH) as a QPI technique (therefore, we replace -QPI by -DH below). Other QPI techniques can also be applied to this concept in general^2,4^. DH and DF-DH are switchable in a single setup by insertion/removal of a DF mask placed in the Fourier plane. A phase-only SLM is put in the sample conjugate plane for phase cancellation^23^. When measuring a sample whose large-OPD structure does not move during the measurement, the SLM input OPD does not need to be refreshed for every measurement, meaning that there is no need to switch the system between DH and DF-DH. Off-axis reference light is illuminated on the image sensor so that the complex field can be measured. The reference light also works as a local oscillator for heterodyne detection to amplify the signal and guarantee shot-noise-limited measurements, which is especially important in PC-DF-DH measurements where the object light is significantly reduced. Note that, to the best of our knowledge, this work is the first demonstration of QPI in the DF configuration (DF-QPI). The specifications of the optical system are given in the Materials and methods and Supplementary Information 1.

### OPD sensitivity of ADRIFT-DH

We theoretically discuss the temporal OPD sensitivity of PC-DF-DH, which determines that of ADRIFT-DH. For simplicity, we assume a case where a transparent sample is illuminated by a plane wave with uniform amplitude distribution *U*_0_. The complex amplitude of the light from the sample arm in the DH configuration at the image sensor can be written as $U_0{\mathrm{e}}^{i\theta _{mn}}$, where *θ*_*mn*_ denotes the OPD map introduced by the sample (*m* and *n* are indices of the image sensor pixels along the *x* and *y* directions, respectively). The intensity at the image sensor in DF imaging with a DC-cut spatial mask placed in the Fourier plane can be approximated as $\vert{U_0({\mathrm{e}}^{i\theta _{mn}^{{\mathrm{PC}}}} - 1)}\vert^2$ when the amount of unscattered light does not largely change with and without the sample. If the maximum OPD in the FOV after phase cancellation is sufficiently small ($\theta _{{\mathrm{max}}}^{{\mathrm{PC}}}$ ≪ 1), then the maximum DF intensity found in the FOV may be described as

1 $$\vert{U_0({\mathrm{e}}^{i\theta _{mn}^{{\mathrm{PC}}}} - 1)} \vert^2 = 2U_0^2\left( {1 - {\mathrm{cos}}\theta _{{\mathrm{max}}}^{{\mathrm{PC}}}} \right)\sim U_0^2\theta _{{\mathrm{max}}}^{{\mathrm{PC}}^2}$$

Therefore, in PC-DF-DH, we can increase the amount of illumination light on the sample by a factor of $1/\theta _{{\mathrm{max}}}^{{\mathrm{PC}}^2}$ because the DH intensity provided by the sample arm is $\left| {U_0{\mathrm{e}}^{i\theta _{mn}}} \right|^2 = U_0^2$ for any sample. The $1/\theta _{{\mathrm{max}}}^{{\mathrm{PC}}^2}$ times stronger illumination light enhances the detected scattered-light intensity, which brings object information, by a factor of $1/\theta _{{\mathrm{max}}}^{{\mathrm{PC}}^2}$. In a holographic measurement, the signal appears in the interferometric term between the sample and reference arm light fields. Since we do not change the amount of light in the reference arm in PC-DF-DH, the signal associated with the scattered-light component is enhanced by $1/\theta _{{\mathrm{max}}}^{{\mathrm{PC}}}$. This allows for a dynamic range shift to the smaller-OPD regime with a $\sim 1/\theta _{{\mathrm{max}}}^{{\mathrm{PC}}}$ times higher signal-to-noise ratio. In addition, there is another, albeit minor, factor of sensitivity improvement: shot-noise reduction by the phase cancellation itself. By cancelling the OPD distribution due to the sample, the amount of light from the sample arm can be reduced to half before cancellation, at most. This factor can reduce the shot noise by a factor of between 1 and 1.4, which depends on the amount of cancelled OPD, in addition to the abovementioned improvement factor of $1/\theta _{{\mathrm{max}}}^{{\mathrm{PC}}}$ from the stronger illumination. Details are discussed in Supplementary Information 2.2.

### Experimental validation of DF-DH

**Fig. 2:**
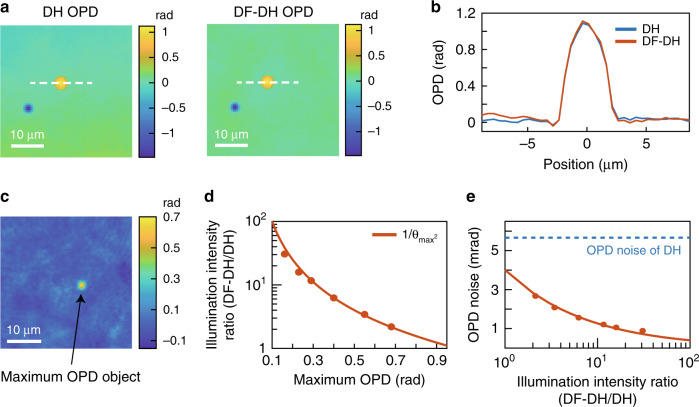
Experimental validation of DF-DH. **a** OPD images measured by DH (left) and DF-DH (right). **b** Cross-sectional profiles along the white dotted lines in (**a**). The blue and orange curves represent the results obtained by DH and DF-DH, respectively. **c** OPD image measured by DF-DH without the sample. An artificial object (indicated by the arrow) with the maximum OPD in the FOV (0.68 rad) is generated with the SLM. The area is used for the sensitivity evaluation. **d** Illumination intensity ratio of DF-DH and DH versus the maximum OPD value in the FOV. The data show good agreement with the theoretical curve (solid line). **e** OPD noise (standard deviation of temporal OPD) versus illumination intensity ratio of DF-DH and DH. The blue dotted line indicates the OPD noise of DH and is shown as a reference. The data show good agreement with the theoretical curve (solid line) described in Supplementary Information 2.3

We provide experimental validation of DF-DH, which is used as a QPI technique in ADRIFT-DH. Figure 2a, b confirm the QPI capability of DF-DH, where the OPD distribution of a 5 μm silica microbead measured by DF-DH shows good agreement with that obtained with conventional DH. The slight global deviation in the two images is likely a high-pass filtering effect caused by the finite size of the DF mask. This effect can be mitigated by using a smaller DF mask. The DF-DH image is reconstructed by using an image of the sample-specific scattered light measured by DF-DH and that of the unscattered light without the sample measured by DH (see Supplementary Information 4 for more details).

We next confirm that a smaller maximum OPD value in the FOV allows us to increase the illumination intensity on the sample. A virtual object with arbitrary OPD value (0.16, 0.23, 0.29, 0.40, 0.55 and 0.68 rad) is created with the SLM (indicated by the arrow in Fig. 2c) and measured with DF-DH by adjusting the illumination intensity on the sample to use the full dynamic range of the image sensor. The illumination intensity ratio of DF-DH to DH as a function of the maximum OPD is plotted in Fig. 2d. The measured data are in good agreement with the theoretical values, ${\mathrm{1/}}\theta _{\max }^2$, derived from Eq. (1).

Finally, we evaluate how the noise (temporal standard deviation of OPD) of DF-DH depends on the intensity of the light illuminating the sample. The noise is evaluated by taking the standard deviation of 20 continuously measured temporal data points acquired at 10 Hz and averaging over 80 × 80 pixels (~40 μm × 40 μm) in Fig. 2c. As Fig. 2e shows, the evaluated data are in good agreement with the theoretical curve (see Supplementary Information 2.3 for more details), and the noise is decreased to 0.9 mrad with a 31 times higher illumination intensity. As a reference, the noise of the DH measurement is also shown in Fig. 2e. It remains at 5.7 mrad for any sample because the illumination light is maintained at the same amount. This is because the OPD produced by a transparent object appears as a spatial shift of the interference fringes rather than as a change in the optical intensity.

### Experimental validation of phase cancellation

**Fig. 3:**
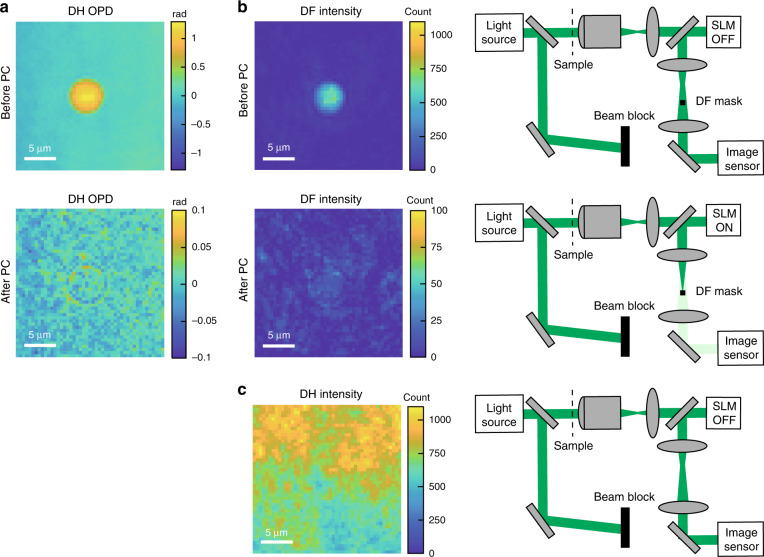
Experimental validation of phase cancellation. **a** DH OPD images before and after phase cancellation. **b** DF intensity images provided by the sample arm before and after phase cancellation. **c** DH intensity image provided by the sample arm

As discussed above, the amount of dynamic range shift to the smaller-OPD regime is determined by the maximum OPD value after phase cancellation. We validate the phase cancellation method by measuring a large-OPD object (5 μm silica microbead), as shown in Fig. 3. Figure 3a shows OPD images measured by DH before and after phase cancellation. The OPD value of the microbead (~1 rad) is well cancelled to <0.1 rad, showing that the phase cancellation concept works. Figure 3b shows DF intensity images of the silica microbead provided by the sample arm before and after phase cancellation. Figure 3c shows an intensity image of the sample arm in DH as a reference. In Fig. 3b, c, the maximum image sensor count is reduced from ~1100 to ~30, a factor of ~35, by switching the system from DH to PC-DF-DH. The comparison clearly shows that the amount of light the image sensor is exposed to is significantly reduced by PC-DF-DH. This enables us to increase the illumination light on the sample and improve the OPD sensitivity.

### Dynamic-range-expanded MIR photothermal QPI of microbeads

**Fig. 4:**
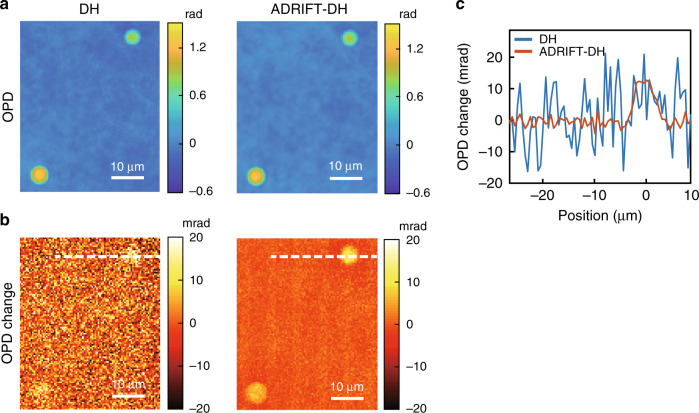
Dynamic-range-expanded MIR photothermal QPI of silica microbeads. **a** OPD images measured by DH (left) and ADRIFT-DH (right) in the MIR OFF state. **b** Images of the photothermal OPD changes due to absorption of the MIR pump light measured by DH (left) and ADRIFT-DH (right). **c** Cross-sectional profiles along the white dotted lines in (**b**). The blue and orange plots represent the results obtained by DH and ADRIFT-DH, respectively

To illustrate the advantage of ADRIFT-QPI, we apply this technique to MIR photothermal QPI^10–13^. MIR photothermal QPI is a recently developed molecular vibrational imaging method, where the refractive index change of the sample caused by the absorption of MIR pump light is detected through the OPD change of visible probe light. In this experiment, silica microbeads immersed in refractive-index-matching oil are used as a sample. The MIR pump pulsed laser is tuned to the wavenumber of 1045 cm^−1^, which is resonant with the O–Si–O stretching mode of silica. Figure 4a shows the pump-OFF-state OPD images obtained by conventional DH and ADRIFT-DH. We can see the same OPD images, including the background surface roughness of the glass plate. The slight deviation between the images comes from the high-pass filtering effect of the DF mask used in ADRIFT-DH discussed in the above section (see Supplementary Information 5 for more details). Figure 4b shows the OPD change (pump ON-OFF) due to absorption of the MIR pump light, and Fig. 4c shows a cross-section of the microbead images. In the ADRIFT-DH measurement, the probe light illumination on the sample is 38 times higher than that in the DH measurement, as the maximum OPD value is decreased to ~0.1 rad by phase cancellation. This reduces the minimum detectable OPD change by a factor of 6.6, which is obtained as a ratio of the noise floor of ADRIFT-DH and that of DH. The noise values are obtained by calculating the spatial standard deviation for part of the FOV where no sample exists in the differential OPD image between the pump ON and OFF states (i.e., Fig. 4b). Note that to achieve the same sensitivity improvement in conventional DH, we have to average 44 images. The small OPD change of a few mrad can be clearly visualized in ADRIFT-DH, which is otherwise buried in the optical shot noise in DH. This demonstration clearly shows the advantage of the expanded dynamic range: the capability of visualizing the original large-OPD (>1 rad) distribution of the sample concurrently with the small OPD changes (~mrad).

### Dynamic-range-expanded MIR photothermal QPI of a live biological cell

**Fig. 5:**
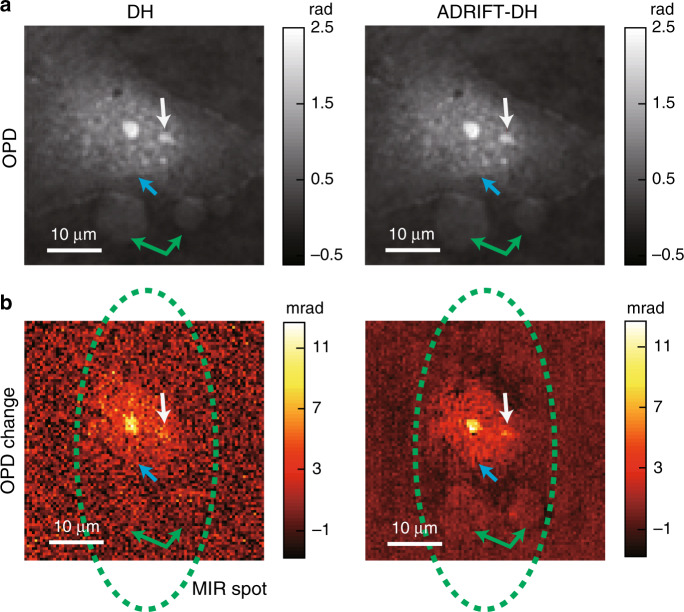
Dynamic-range-expanded MIR photothermal QPI of a live COS7 cell. **a** OPD images measured by DH (left) and ADRIFT-DH (right) in the MIR OFF state. The white, blue and green arrows show the existence of the nucleoli, nucleus and two particles, respectively. **b** Images of the photothermal OPD changes due to absorption of the MIR pump light measured by DH (left) and ADRIFT-DH (right). The area in the green dotted circle indicates the illumination spot of the MIR pump light. The structures indicated by the white (nucleoli), blue (nucleus) and green (particles) arrows in (**a**) also give stronger signals in the photothermal images shown in (**b**), which is visualized clearly with ADRIFT-DH but not with DH

As a more practical demonstration, we show dynamic-range-expanded MIR photothermal QPI of a live biological cell. The MIR pump light is tuned to 1550 cm^−1^, which is resonant with the peptide bond amide II band mainly found in proteins. Figure 5a shows the pump-OFF-state OPD images obtained by DH and ADRIFT-DH. We decrease the maximum OPD value in the FOV to ~0.1 rad by phase cancellation and increase the probe illumination light by a factor of 17, which is limited by the laser power in this particular case. The illumination light can be further increased with a proper light source to make full use of the sensor dynamic range. The increased illumination light reduces the minimum detectable OPD change by a factor of 3.7, which corresponds to decreasing the averaging number by a factor of 14 compared to conventional DH. Figure 5b shows the OPD change between the ON and OFF states due to absorption of the MIR pump light. Only ADRIFT-DH clearly visualizes the signal localization, especially at the nucleus, nucleoli and some particles indicated by the arrows in Fig. 5a, which could represent the richness of proteins^13^.

## Discussion

The conceptual essence of ADRIFT-QPI is not only the use of stronger illumination but also the implementation of the “DF configuration” and “phase cancellation”, which enables “adaptive” dynamic range expansion to be achieved throughout the entire FOV irrespective of the sample OPD distribution. This advantage differentiates ADRIFT-QPI from other existing techniques that also enable acquisition of high-dynamic-range OPD images with DH, where only the exposure condition (or equivalently, the illumination intensity) is varied. Specifically, a technique to obtain a high-SNR hologram synthesized from multiple holograms recorded with different exposure conditions (i.e., over- and under-exposure) exists^24^. This technique improves the OPD sensitivity only at dark pixels (i.e., pixels where the light intensity is lower than that in other regions of the FOV), but the improved sensitivity is still limited by the sensor saturation capacity. When a transparent sample, such as a biological cell, is observed by this technique, the OPD sensitivity improvement due to over-exposure (or, equivalently, stronger illumination) can be effective only in limited regions of the hologram (i.e., in “valleys” of the interferometric pattern of the hologram), while other regions saturate, preventing dramatic OPD dynamic range expansion. Meanwhile, this technique could achieve further OPD sensitivity improvement if combined with DF-DH. However, the sensitivity improvement factor at each pixel in the FOV depends on the OPD value of the object that exists there. In the DF configuration, a larger-OPD object appears as a brighter spot at the sensor, which allows the use of over-exposure (or equivalently, stronger illumination) without sensor saturation at pixels where only small-OPD objects exist. Note that this can increase the OPD sensitivity at these specific locations but not at other pixels where larger-OPD objects exist because sensor saturation occurs there. Therefore, significant sensitivity improvement cannot be achieved at locations where large-OPD objects, such as biological cells, exist. On the other hand, our ADRIFT-QPI, which works through a combination of the “DF configuration” and “phase cancellation”, allows for “adaptive” dynamic range expansion regardless of the OPD distribution of the sample, which allows for detection of small OPD changes on top of large-OPD objects. The “phase cancellation” mostly cancels the OPD distribution of the sample and reduces the brightness of the large-OPD objects in the DF image. This allows us to significantly increase the illumination light (or equivalently, to perform over-exposure) at these pixels without sensor saturation. Consequently, adaptive OPD sensitivity enhancement can be achieved throughout the entire FOV irrespective of the sample OPD distribution.

The amount of dynamic range shift (expansion) can be limited by two factors. One is the maximum OPD after phase cancellation, and the other is the amount of increased light the sample is exposed to. In our experiment, the OPD was cancelled down to 0.1 rad, which allows us to increase the illumination light by a factor of 100, but in reality, it was limited to 38 because of the imperfection of the DF filtering due to wavefront distortion of the illumination light of the system, which can be mitigated with careful implementation of the system. The OPD can be further cancelled by improving the alignment of the SLM with respect to the magnified image of the sample. Theoretically, an 8-bit SLM allows for OPD cancellation down to 0.025 rad such that ~1000 times larger illumination, hence ~33 times higher sensitivity, is achievable. A larger-bit SLM would even improve it, although the SLM noise would eventually be the limitation.

In our demonstration of ADRIFT-DH, it is necessary to switch the system from DH to DF-DH, which limits the temporal resolution. However, we believe that there are several strategies to overcome this limitation and perform higher-speed imaging. First, an electrical switching device, such as a digital micromirror device, could be used to achieve fast switching between DH and DF-DH at a kHz rate. Second, we can avoid the switching itself by the following two methods. In one, we can separately implement DH and DF-DH with multiple image sensors. In the other, we measure all the necessary images by DF-DH if the reconstruction algorithm and calculation speed can be optimized (i.e., there is no need to use DH in the first measurement). Third, the SLM input OPD values do not need to be refreshed for every measurement because the large-OPD structures of a cell, which we want to cancel out, do not move fast. We can capture the quick movement of small particles inside the cell without refreshing the SLM pattern because these OPD changes are small enough to be measured only by DF-DH.

It is important to consider photodamage to biological samples because the illumination intensity of ADRIFT-QPI may be increased by dozens of times compared to conventional QPI. However, the illumination intensity used in QPI is generally much lower than that used in other live-cell imaging techniques such as fluorescence and Raman imaging. Therefore, increasing the illumination light with our ADRIFT-QPI may not result in a significant drawback compared to other imaging modalities. For example, in our work, the illumination intensity ~1 nW/μm^2^, which is 2 and 6–8 orders of magnitude lower than that used in fluorescence^25^ and Raman^26^ imaging, respectively. We should also note that scattering (i.e., not absorption) measurements basically lead to less photodamage than fluorescence imaging even under the same illumination intensity. Even if we use a higher-speed image sensor with a kHz frame rate, which requires a 2 orders of magnitude larger amount of light illumination, the intensity is still 4–6 orders of magnitude lower than that used in Raman imaging. Furthermore, the current optical system has room for improvement in terms of optical throughput. For example, by placing the SLM before the sample, an ~5 times reduction in the illumination intensity can be achieved.

The required specifications of an image sensor suitable for ADRIFT-QPI are described as follows. Since active illumination can be used to nearly saturate the image sensor in QPI, the dynamic range is determined by the optical shot noise. Therefore, the sensor read-out noise is not very important as long as it is low enough to be ignored compared to the optical shot noise. This means that a low-read-out-noise sensor such as an sCMOS sensor is not required. Indeed, we use a typical CMOS image sensor with a 10,000 e^−^/pixel full well capacity and 10 e^−^/pixel read-out noise. On the other hand, the sensor full well capacity is important in improving the shot-noise-limited SNR. An image sensor with ultrahigh full well capacity^27^ (e.g., Q-2HFW from Adimec) can be used to further improve the sensitivity of ADRIFT-QPI. Although the illumination intensity further increases with the use of this special sensor, it is 3–5 orders of magnitude lower than that used in Raman imaging and reasonable for live-cell imaging.

The dynamic range can be better shifted in PC-DF-DH by implementing amplitude cancellation in addition to phase cancellation (see Supplementary Information 3 for more details). This is especially useful for measuring non-transparent and/or defocused samples (e.g., thick or overlapped samples) because the amplitude distribution causes inefficient DF rejection.

In this work, we used ADRIFT-QPI to improve MIR photothermal QPI, but it can also be used for other applications. For example, there are some situations where the substrate static roughness can be decoupled from the signal, such as in flow cytometry^28^, optical tweezer applications^29^, optical diffraction tomography^30–32^, and detection of dynamic OPD changes^6,9,12^. In addition, due to the capability to adaptively shift the dynamic range regardless of the sample condition, ADRIFT-QPI has the potential to be as sensitive as the state-of-the-art wide-field scattering imaging techniques, such as iSCAT, which is used for small particle measurement with extremely high sensitivity, even in the presence of highly scattering objects. We note that ADRIFT-QPI can be understood as a forward scattering counterpart of backscattering-based iSCAT. Therefore, it could provide a new opportunity to study the behaviour of small particles inside and outside cells without losing cellular morphological information. The MIR photothermal QPI technique can also be implemented in the same system to add molecular contrast.

## Materials and methods

### Light source

The visible light source is based on second harmonic generation (SHG) of a 10-ns, 1000-Hz, 1064-nm pulsed Q-switched laser (NL204-1K, Ekspla) with a nonlinear crystal LBO (Eksma Optics). The spatial mode of the SHG beam is cleaned by a single-mode optical fibre (P3-405B-FC-5, Thorlabs). The spectral bandwidth is ~2 nm after the fibre, which reduces the coherent noise. We note that a CW laser can be used for many applications of this technique, although a nanosecond pulsed laser is required as the probe light for MIR photothermal QPI.

### ADRIFT-DH system

A complete description of the optical system is provided in Supplementary Information 1. Linearly polarized light is created by a polarizer, and its polarization direction is precisely adjusted by a half-wave plate to the orientation of the liquid crystals in the SLM. The light is split into two by a beamsplitter (BS061, Thorlabs). In the sample arm, the intensity of the illumination light can be adjusted with a neutral density (ND) filter (NDC-50C-2-A, Thorlabs) placed before the sample. The sample image is magnified by a factor of 44 at the image sensor (acA2440-75 μm, Basler) with an objective lens with an NA of 0.6 (LUCPLFLN40X, Olympus) and relay lenses (AC508-100-A and AC508-200-A, Thorlabs). The image sensor (acA2440-75 μm, Basler) has a full well capacity of ~10,000 e^−^/pixel. A phase-only SLM [1920 × 1152 XY Phase Series Spatial Light Modulator (Meadowlark Optics)] is placed in the sample conjugate plane. A circular mask deposited on a glass substrate (50 or 100 μm in diameter, TOPRO) is put in the Fourier plane as a DF mask. In the reference arm, a delay line and a beam expander (A397TM-A and AC254-075-A, Thorlabs) adjust the differences in the optical path length and beam diameter between the two arms, respectively. A transmission grating with 100 line-pairs/mm (66–341, Edmund Optics) placed in the sample conjugate plane and two lenses (AC508-100-A, Thorlabs) create off-axis reference light. The laser intensity fluctuation (1–2% in our case) is numerically compensated by recording a part of the light with another camera (acA2440-75 μm, Basler) so that shot-noise-limited detection is achieved. We mitigate background OPD fluctuations caused by convection of the ambient air by enclosing the system in a box. The image sensor is operated at 10 or 20 Hz with an exposure time of 60 or 30 ms for the experiments shown in Figs. 2–5, respectively. The number of pixels is reduced from 1024 × 1024 (raw interferogram) to 152 × 152 (complex-field reconstruction) through the phase retrieval process described in Supplementary Information 2.1. The reconstructed image has a diffraction-limited pixel size of ~500 nm. The visible illumination power at the sample plane can be increased up to ~100 μW.

### Phase cancellation

The phase cancellation requires the following calibration for estimating the voltages to be loaded on each pixel of the SLM (consisting of N pixels) from the OPD image measured with DH (consisting of M pixels), where the number of pixels generally does not match between the two images, with N > M. We generate a set of calibration images that links the N-pixel SLM image and M-pixel OPD image for each of the 256 (8 bit) phase gradients of the SLM. This can be made by inputting a uniform voltage to all N pixels of the SLM and measuring the corresponding M-pixel OPD image by DH. Then, by using the set of calibration images, we translate the measured M-pixel OPD image to the N-pixel SLM input voltage image for phase cancellation. In case the phase cancellation does not sufficiently work, we can iteratively run the cancellation procedure with feedback on the uncancelled remaining OPD distribution.

### Samples

Porous silica microbeads [43-00-503, Sicastar (micromod Partikeltechnologies GmBH)] immersed in index-matching oil (refractive index 1.50 at 587.56 nm, Shimadzu) are used as the sample for the experiments shown in Figs. 2–4. The COS7 cells (Riken) for the experiment shown in Fig. 5 are cultured in Dulbecco’s modified Eagle’s medium (DMEM) with 10% foetal bovine serum supplemented with penicillin–streptomycin, L-glutamine, sodium pyruvate and nonessential amino acids at 37 °C in 5% CO_2_. For live-cell imaging, the cells are cultured in a 35-mm glass-bottomed dish (AGC Techno Glass), and the medium is replaced by phenol red-free culture medium containing HEPES buffer (2 mL) before imaging. All solutions are from Thermo Fisher Scientific.

### MIR photothermal QPI of microbeads

MIR pulses with a duration of 5 μs lasing at 1045 cm^−1^ provided by a quantum cascade laser (QD9500CM1, Thorlabs) are used as the pump light. A ZnSe lens (LA7733-G, Thorlabs) with a focal length of 20 mm is used to loosely focus the MIR light onto the sample with an excitation-field diameter of ~75 μm. The MIR ON-OFF modulation rate is 5 Hz. The MIR pulse energy at the sample plane is ~50 nJ. The diameter of the DF mask is 100 μm.

### MIR photothermal QPI of a COS7 cell

MIR pulses with a duration of 1 μs lasing at 1550 cm^−1^ provided by a quantum cascade laser [DO418, Hedgehog (Daylight Solutions)] are used as the pump light. A ZnSe lens (LA7733-G, Thorlabs) with a focal length of 20 mm is used to loosely focus the MIR light onto the sample with excitation-field elliptical major and minor axes of ~70 and ~30 μm, respectively. The MIR pulse energy at the sample plane is ~100 nJ. The diameter of the DF mask is 50 μm.

## Supplementary information

Supplementary Information for Adaptive dynamic range shift (ADRIFT) quantitative phase imaging

## Data Availability

The data provided in the manuscript and supplementary information are available from T.I. upon request.
