# Supplementary material for: Adaptive dynamic range shift (ADRIFT) quantitative phase imaging
Source: Light Sci Appl. 2021 Jan 1;10:1. doi: 10.1038/s41377-020-00435-z (PMC7775917; doi:10.1038/s41377-020-00435-z)
Supplement: Supplementary file 1 — Supplementary Information for Adaptive dynamic range shift (ADRIFT) quantitative phase imaging [file 41377_2020_435_MOESM1_ESM.docx]

**Supplementary Information:**

**Adaptive dynamic range shift (ADRIFT) quantitative phase imaging**

Keiichiro Toda,^1^ Miu Tamamitsu,^1^ and Takuro Ideguchi^1,2,3,*^

^1^Department of Physics, The University of Tokyo, Tokyo 113-0033, Japan

^2^Institute for Photon Science and Technology, The University of Tokyo, Tokyo 113-0033, Japan

^3^PRESTO, Japan Science and Technology Agency, Saitama 332-0012, Japan

^*^Corresponding author: [ideguchi@ipst.s.u-tokyo.ac.jp](mailto:ideguchi@ipst.s.u-tokyo.ac.jp)

1. **Optical implementation of ADRIFT-DH**

The complete description of the ADRIFT-DH system is shown in Fig. S1.

**
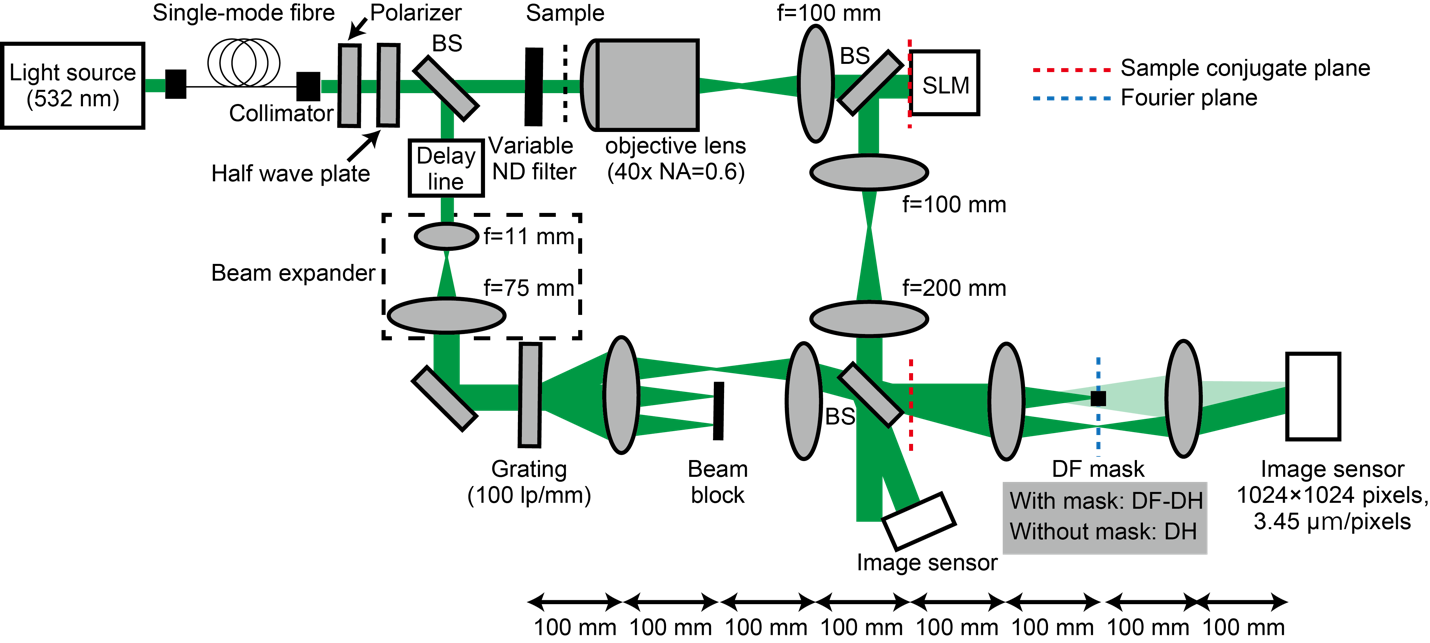
**

**Fig. S1 | Complete description of the ADRIFT-DH system.**

1. **Optical phase delay (OPD) sensitivity of ADRIFT-DH**
   1. **OPD extraction procedure**

First, we explain how to extract quantitative optical phase delay (OPD) map by off-axis digital holography (DH) and phase-cancelling dark-field off-axis digital holography (PC-DF-DH). For simplicity, we assume a case where a transparent sample is illuminated by a plane wave with uniform amplitude distribution $\text{U}_{\text{0}}$. We denote OPD maps introduced by the sample with and without phase cancellation as $\theta_{mn}^{\mathrm{PC}}$ and $\theta_{mn}$ (where $m\in\left[ 0, M-1 \right]$ and $n\in\left[ 0, N-1 \right]$ are pixel-indices of the image-sensor along *x* and *y* directions, respectively), respectively, and the maximum OPD in the field of view (FOV) with phase cancellation as $\theta_{max}^{\mathrm{PC}} \left( \ll1 \right)$. In PC-DF-DH, we assume that amount of illumination light on the sample is $\alpha<1/{\left( \theta_{max}^{\mathrm{PC}} \right)^{2}}$ times higher than that in DH. We also assume to use a reference optical field for the off-axis holographic detection that has the same uniform amplitude distribution as that illuminating the sample (i.e.,$U_{0}$), maximizing the interferometric visibility^1^. Intensity at the image sensor in DH,$I_{mn}^{\mathrm{DH}}$, and PC-DF-DH,$I_{mn}^{PC-DF-DH}$, can be described, respectively, as

　　$I_{mn}^{\mathrm{DH}}\text{=}\left| U_{0}e^{i\theta_{mn}}+U_{0}e^{i\left( k_{M}m+k_{N}n \right)} \right|^{2}=2\left| U_{0} \right|^{2}+\left| U_{0} \right|^{2}e^{i\theta_{mn}}e^{-i\left( k_{M}m+k_{N}n \right)}+c.c.$ (S1)

and

$$I_{mn}^{PC-DF-DH}\text{\textasciitilde}\left| {\sqrt{\alpha}U}_{0}\left( e^{i\theta_{mn}^{PC}}-1 \right)+U_{0}e^{i\left( k_{M}m+k_{N}n \right)} \right|^{2}$$

$\sim\left| U_{0} \right|^{2}+\alpha{\left| U_{0} \right|^{2}\left( \theta_{mn}^{\mathrm{PC}} \right)}^{2}+\sqrt{\alpha}\left| U_{0} \right|^{2}\left( e^{i\theta_{mn}^{\mathrm{PC}}}-1 \right)e^{-i\left( k_{M}m+k_{N}n \right)}+c.c.$, (S2)

where $\left( k_{M},k_{N} \right)$ represents the off-axis wavevector of the reference field. The approximation in Eq. (S2) is valid when (1) the sample is sparse and/or has small-OPD distribution and (2) the DF-filtering mask is sufficiently small so that the spatial DC component rejected by the DF mask can be represented by ${\sqrt{\alpha}U}_{0}$. We are interested in the complex-amplitude information found in the cross-terms in Eqs. (S1) and (S2), i.e., $\left| U_{0} \right|^{2}e^{i\theta_{mn}}$ and $\sqrt{\alpha}\left| U_{0} \right|^{2}\left( e^{i\theta_{mn}^{\mathrm{PC}}}-1 \right)$, respectively. The cross-term can be extracted through the computational procedure summarized in Fig. S2, which consists of (1) 2D discrete Fourier transformation, (2) sideband centering, (3) low-pass (LP) filtering and (4) 2D inverse discrete Fourier transformation. The extracted complex amplitudes can be described as

$E_{mn}^{\mathrm{DH}}=LP\left[ I_{mn}^{\mathrm{DH}}e^{i\left( k_{M}m+k_{N}n \right)} \right]=\left| U_{0} \right|^{2}e^{i\theta_{mn}}$ (S3)

and

$E_{mn}^{PC-DF-DH}=LP\left[ I_{mn}^{PC-DF-DH}e^{i\left( k_{M}m+k_{N}n \right)} \right]=\sqrt{\alpha}\left| U_{0} \right|^{2}\left( e^{i\theta_{mn}^{\mathrm{PC}}}-1 \right)$, (S4)

where the sideband centering is performed by multiplying $e^{i\left( k_{M}m+k_{N}n \right)}$ to the interferogram and the LP-filtering (indicated by the operator $\mathrm{LP}$ in the equations) is performed with a pupil function $H_{kl}$ (where $k\in\left[ 0, M-1 \right]$ and $l\in\left[ 0, N-1 \right]$ are indices in the spatial-frequency domain). The pupil function is typically a binary circular aperture whose radius is determined by the numerical aperture of the objective lens. Finally, the corresponding OPD maps, $\theta_{mn}$ and $\theta_{mn}^{\mathrm{PC}}$, can be obtained as

$\theta_{mn}\text{ = }\angle\text{ [}{E_{mn}^{\mathrm{DH}}}/{\left| U_{0} \right|^{2}}\text{]}$ (S5)

and

$\theta_{mn}^{\mathrm{PC}}=\angle\left[ {{E_{mn}^{PC-DF-DH}}/{\sqrt{\alpha}\left| U_{0} \right|}}^{2}+1 \right]$, (S6)

where $\angle$ denotes phase extraction operation.


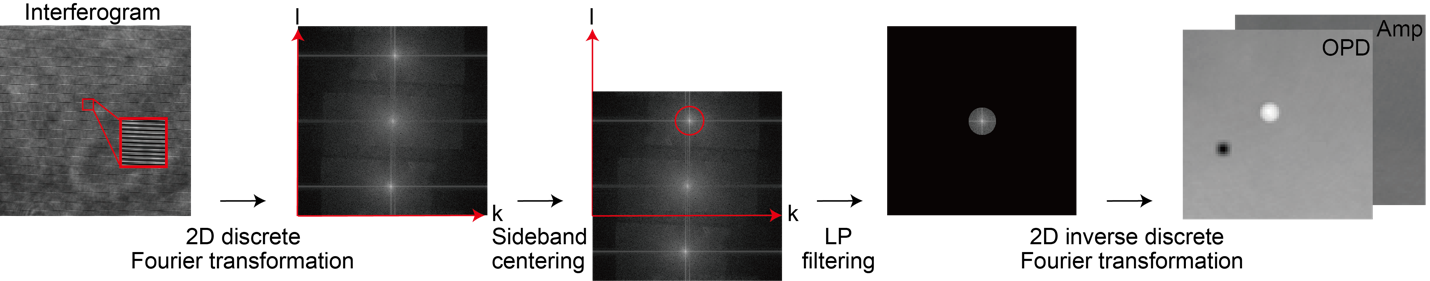


**Fig. S2 | Computational procedure to extract the cross term from the measured interferogram.** The $k\in\left[ 0, M-1 \right]$ and $l\in\left[ 0, N-1 \right]$ represent wavenumbers in the spatial-frequency domain.

- 1. **OPD sensitivity**

The OPD sensitivity of adaptive dynamic range shift digital holography (ADRIFT-DH) is determined by the sensitivity of PC-DF-DH. To clarify the sensitivity improvement achieved by the ADRIFT method, we theoretically compare the temporal OPD sensitivity of DH and PC-DF-DH. Our calculation follows the analysis reported in ref. [1]. We begin with adding noise terms to the noise-free interferograms represented by Eqs. (S1) and (S2), such that they are modified as

$\acute{I}_{mn}^{\mathrm{DH}}=\left| U_{0}e^{i\theta_{mn}}+U_{0}e^{i\left( k_{M}m+k_{N}n \right)} \right|^{2}+Z_{mn}^{\mathrm{DH}}$ (S7)

and

$\acute{I}_{mn}^{PC-DF-DH}\sim\left| \sqrt{\alpha}U_{0}\left( e^{i\theta_{mn}^{\mathrm{PC}}}-1 \right)+U_{0}e^{i\left( k_{M}m+k_{N}n \right)} \right|^{2}+Z_{mn}^{PC-DF-DH}$

$\sim\left| U_{0} \right|^{2}\left| i\theta_{mn}^{\mathrm{PC}}\sqrt{\alpha}+e^{i\left( k_{M}m+k_{N}n \right)} \right|^{2}+Z_{mn}^{PC-DF-DH}$, (S8)

where $Z_{mn}^{\mathrm{DH}}$ and $Z_{mn}^{PC-DF-DH}$ represent the noise. Here we consider that the optical shot noise is the dominant noise source assuming the interferometer is sufficiently stable^1^. The OPD images $\acute{\theta}_{mn}$ and $\acute{\theta}_{mn}^{\mathrm{PC}}$ can be reconstructed by replacing $I_{mn}^{\mathrm{DH}}$ and $I_{mn}^{PC-DF-DH}$ in Eqs. (S3) and (S4) by $\acute{I}_{mn}^{\mathrm{DH}}$ and $\acute{I}_{mn}^{PC-DF-DH}$, so that

$\acute{\theta}_{mn}=\angle\left[ {\mathrm{LP}\left\{ \acute{I}_{mn}^{\mathrm{DH}}e^{i\left( k_{M}m+k_{N}n \right)} \right\}}/{\left| U_{0} \right|^{2}} \right]=\angle\left[ {e^{i\theta_{mn}}+LP\left\{ Z_{mn}^{\mathrm{DH}}e^{i\left( k_{M}m+k_{N}n \right)} \right\}}/{\left| U_{0} \right|^{2}} \right]$

$=\angle\left[ e^{i\theta_{mn}}\left( 1+\frac{\mathrm{LP}\left\{ Z_{mn}^{\mathrm{DH}}e^{i\left( k_{M}m+k_{N}n \right)} \right\}}{\left| U_{0} \right|^{2}e^{i\theta_{mn}}} \right) \right]⋍\theta_{mn}+\frac{Z_{mn}^{\mathrm{DH}}\sin\left( k_{M}m+k_{N}n-\theta_{mn} \right)\bigotimes h_{mn}}{\left| U_{0} \right|^{2}}$ (S9)

and

$\acute{\theta}_{mn}^{\mathrm{PC}}=\angle\left[ {\mathrm{LP}\left\{ \acute{I}_{mn}^{PC-DF-DH}e^{i\left( k_{M}m+k_{N}n \right)} \right\}}/{{\sqrt{\alpha}\left| U_{0} \right|}^{2}}+1 \right]=\angle\left[ {e^{i\theta_{mn}^{\mathrm{PC}}}+LP\left\{ Z_{mn}^{PC-DF-DH}e^{i\left( k_{M}m+k_{N}n \right)} \right\}}/{\sqrt{\alpha}\left| U_{0} \right|^{2}} \right]$

$=\angle\left[ e^{i\theta_{mn}^{\mathrm{PC}}}\left( 1+\frac{\mathrm{LP}\left\{ Z_{mn}^{PC-DF-DH}e^{i\left( k_{M}m+k_{N}n \right)} \right\}}{{\sqrt{\alpha}\left| U_{0} \right|}^{2}e^{i\theta_{mn}^{\mathrm{PC}}}} \right) \right]⋍\theta_{mn}^{\mathrm{PC}}+\frac{Z_{mn}^{PC-DF-DH}\sin\left( k_{M}m+k_{N}n-\theta_{mn}^{\mathrm{PC}} \right)\bigotimes h_{mn}}{\sqrt{\alpha}\left| U_{0} \right|^{2}}$, (S10)

where $h_{mn}$ is the inverse Fourier transformation of $H_{kl}$ and $\bigotimes$ is the convolution operator. We define the OPD sensitivity by the temporal OPD standard deviation at each spatial pixel which we denote by $\delta\acute{\theta}_{mn}^{\mathrm{DH}}$ and $\delta\acute{\theta}_{mn}^{PC-DF-DH}$　for DH and PC-DF-DH, respectively. They can be obtained by calculating square root of variance (Var) of $\acute{\theta}_{mn}$ and $\acute{\theta}_{mn}^{\mathrm{PC}}$ as

$\delta\acute{\theta}_{mn}^{\mathrm{DH}}=\sqrt{\mathrm{Var}\left( \acute{\theta}_{mn} \right)}=\frac{1}{\left| U_{0} \right|^{2}}\sqrt{\left\{ \mathrm{Var}\left( Z_{mn}^{\mathrm{DH}} \right)\sin^{2} \left( k_{M}m+k_{N}n-\theta_{mn} \right) \right\}\bigotimes{h_{mn}}^{2}}$

$=\frac{1}{\left| U_{0} \right|^{2}}\sqrt{\left\{ \mathrm{Var}\left( Z_{mn}^{\mathrm{DH}} \right)\frac{1-\cos2\left( k_{M}m+k_{N}n-\theta_{mn} \right)}{2} \right\}\bigotimes{h_{mn}}^{2}}$ (S11)

and

$\delta\acute{\theta}_{mn}^{PC-DF-DH}=\sqrt{\mathrm{Var}\left( \acute{\theta}_{mn}^{\mathrm{PC}} \right)}=\frac{1}{\sqrt{\alpha}\left| U_{0} \right|^{2}}\sqrt{\left\{ \mathrm{Var}\left( Z_{mn}^{PC-DF-DH} \right)\sin^{2} \left( k_{M}m+k_{N}n-\theta_{mn}^{\mathrm{PC}} \right) \right\}\bigotimes{h_{mn}}^{2}}$

$=\frac{1}{\sqrt{\alpha}\left| U_{0} \right|^{2}}\sqrt{\left\{ \mathrm{Var}\left( Z_{mn}^{PC-DF-DH} \right)\frac{1-\cos2\left( k_{M}m+k_{N}n-\theta_{mn}^{\mathrm{PC}} \right)}{2} \right\}\bigotimes{h_{mn}}^{2}}$. (S12)

Here we remember that the variance of Poisson distribution (which is the case of the shot noise) is equal to the mean of the signal. Therefore, the following relations hold:

$\mathrm{Var}\left( Z_{mn}^{\mathrm{DH}} \right)=Mean \left( \acute{I}_{mn}^{\mathrm{DH}} \right)=2\left| U_{0} \right|^{2}\left\{ 1+\cos\left( \theta_{mn}-k_{M}m-k_{N}n \right) \right\}$, (S13)

$\mathrm{Var}\left( Z_{mn}^{PC-DF-DH} \right)=Mean \left( \acute{I}_{mn}^{PC-DF-DH} \right)\sim\left| U_{0} \right|^{2}\left\{ 1+\alpha\left( \theta_{mn}^{\mathrm{PC}} \right)^{2}+2\sqrt{\alpha}\theta_{mn}^{\mathrm{PC}}\sin\left( k_{M}m+k_{N}n \right) \right\}$, (S14)

where $\mathrm{Mean}$ calculates the mean of the argument. All terms outside the LP bandwidth such as $\cos2\left( k_{M}m+k_{N}n-\theta_{mn} \right)$ in Eqs. (S11) and (S12),$\cos\left( \theta_{mn}-k_{M}m-k_{N}n \right)$ in Eq. (S13) and $\sin\left( k_{M}m+k_{N}n \right)$ in Eq. (S14) can be removed by the LP-filtering operation $\bigotimes{h_{mn}}^{2}$. Since $H_{kl}$ is unity within its passband and zero elsewhere, ${h_{mn}}^{2}$ can be approximated as the delta function $\delta_{mn}$, and the summation of its amplitude over the whole pixels is $\sum_{mn} {h_{mn}}^{2}=\sum_{mn} {\left| H_{kl} \right|^{2}}/{MN}=R/{MN}$, where $R$ is the aperture area of $H_{kl}$. By inserting Eqs. (S13) and (S14) into Eqs. (S11) and (S12), respectively, we can obtain

$\delta\acute{\theta}_{mn}^{\mathrm{DH}}=\frac{\sqrt{\left| U_{0} \right|^{2}\bigotimes{h_{mn}}^{2}}}{\left| U_{0} \right|^{2}}\sim\frac{1}{\left| U_{0} \right|}\sqrt{\frac{R}{MN}}$ (S15)

and

$\delta\acute{\theta}_{mn}^{PC-DF-DH}=\frac{\sqrt{\left| U_{0} \right|^{2}\left\{ 1+\alpha\left( \theta_{mn}^{\mathrm{PC}} \right)^{2} \right\}\bigotimes{h_{mn}}^{2}}}{\sqrt{2\alpha}\left| U_{0} \right|^{2}}\sim\frac{\sqrt{1+\alpha\left( \theta_{mn}^{\mathrm{PC}} \right)^{2}}}{\sqrt{2\alpha}\left| U_{0} \right|}\sqrt{\frac{R}{MN}}=\frac{\sqrt{\beta_{mn}}}{\sqrt{\alpha}}\delta\acute{\theta}_{mn}^{\mathrm{DH}}$. (S16)

Here, we define $\beta_{mn}=\left\{ 1+\alpha\left( \theta_{mn}^{\mathrm{PC}} \right)^{2} \right\}/2$, which corresponds to the ratio of the number of photons that contributes to the OPD reconstruction between PC-DF-DH and DH [refer to Eqs. (S7) and (S8). The sum of the intensity of the sample and reference arms in PC-DF-DH and DH are $\left| U_{0} \right|^{2}\left\{ \alpha\left( \theta_{mn}^{\mathrm{PC}} \right)^{2}+1 \right\}$ and $2\left| U_{0} \right|^{2}$, respectively]. As described in the main text, Eq. (S16) describes the main ($\sqrt{\alpha}$) and minor ($1/\sqrt{\beta_{mn}}$) factors for the sensitivity improvement in ADRIFT-DH. The main factor is due to the shift of the dynamic range to the smaller OPD regime, which is the result of the increase of the illumination light onto the sample (hence the detected photon flux of the sample-specific scattered field). The minor factor is due to the reduction of the optical shot noise when $\theta_{mn}^{\mathrm{PC}}<\theta_{max}^{\mathrm{PC}}$ because the DF intensity is proportional to ${\theta_{mn}^{\mathrm{PC}}}^{2}$.

- 1. **Experimental validation**

We validate our experimental results of the sensitivity improvement shown in Fig. 2e in the main text with Eq. (S16). We can approximate $\beta\sim0.5$ in the region in Fig. 2c in the main text, since $\alpha\left( \theta_{mn}^{\mathrm{PC}} \right)^{2}\ll1$. Therefore, the equation can be simplified as $\delta\acute{\theta}_{mn}^{PC-DF-DH}\sim{\delta\acute{\theta}_{mn}^{\mathrm{DH}}}/\sqrt{2\alpha}$ which shows good agreement with the data points for various experimental values of $\alpha$ as shown in Fig. 2e in the main text.

1. **Effect of the amplitude distribution of the optical field on phase cancellation**

We discuss how the existence of the amplitude distribution of the optical field (created by, for example, light-absorption by non-transparent sample, diffraction effect of defocused objects, etc.) can limit the reduction of the DF intensity after phase cancellation. For simplicity, we assume a case where the sample is illuminated by a plane wave with uniform amplitude distribution $U_{0}$. DF intensity at the image sensor after phase cancellation can be approximated by

$\left| U_{mn}e^{i\theta_{mn}^{\mathrm{PC}}}-U_{0} \right|^{2}\sim\left| U_{mn}-U_{0} \right|^{2}+U_{mn}U_{0}\left( \theta_{mn}^{\mathrm{PC}} \right)^{2}$ (S17)

where $U_{mn}$ and $\theta_{mn}^{\mathrm{PC}}$ are the amplitude and OPD maps after phase cancellation ($\theta_{mn}^{\mathrm{PC}}$<< 1), respectively. From Eq. (S17), we find that the value of $\left| U_{mn}-U_{0} \right|^{2}$ in the FOV can be larger than $U_{mn}U_{0}\left( \theta_{mn}^{\mathrm{PC}} \right)^{2}$, thereby limiting the DF rejection rate even if the OPD distribution is well cancelled ($\theta_{mn}^{\mathrm{PC}}\sim0$). It ends up with the less increase of the illumination intensity on the sample. This limitation can be solved by implementing amplitude cancellation in addition to phase cancellation.

1. **Reconstruction procedure of the OPD map with dark-field digital holography (DF-DH)**

We describe how to reconstruct an OPD image by DF-DH when the imaging object is sparse and/or has small-OPD distribution. In practice, an illumination optical field is not a perfect plane wave. We denote the complex amplitude of the light before and after interacting with the sample as $U_{mn}^{\mathrm{in}}e^{i\psi_{mn}}$and $U_{mn}e^{i\left[ \theta_{mn}+\psi_{mn} \right]}$, respectively, where $\theta_{mn}$ and ${U_{mn}}/{U_{mn}^{\mathrm{in}}}$ are the OPD and amplitude distributions introduced by the sample, respectively. The reference field is written as $R_{mn}e^{i\phi_{mn}}$. We consider that the DF mask is sufficiently small to reject the plane-wave component denoted by $U_{00}$. We assume that this spatial DC component of the illumination field ($U_{00}$) is mostly unaltered by the presence of the transparent sample when it is sparse and/or has small-OPD distribution. Note that this condition can be always met after phase cancellation of transparent object, but can also be true without phase cancellation when imaging, e.g., a sparse sample such as the microbead sample shown in Fig. 2 in the main text. The interferometric term measured by DF-DH with the sample ($E_{mn}^{DF-DH}$) and that measured by DF-DH and DH without the sample ($E_{mn}^{DF-DH\_bg}$ and $E_{mn}^{DH\_bg}$, respectively) can be described as

$E_{mn}^{DF-DH}=\left\{ \sqrt{a}\left( U_{mn}e^{i\left[ \theta_{mn}+\psi_{mn} \right]}-U_{00} \right) \right\}\left( R_{mn}e^{i\phi_{mn}} \right)^{*}$, (S18)

$E_{mn}^{DF-DH\_bg}=\{\sqrt{a}\left( U_{mn}^{\mathrm{in}}e^{i\psi_{mn}}-U_{00} \right)\}\left( R_{mn}e^{i\phi_{mn}} \right)^{*}$ (S19)

and

$E_{mn}^{DH\_bg}=\left( U_{mn}^{\mathrm{in}}e^{i\psi_{mn}} \right)\left( R_{mn}e^{i\phi_{mn}} \right)^{*}$, (S20)

where * represents the complex conjugate. We assume that intensity of the light illuminating the sample is $a$ times higher in DF-DH than that in DH. Note that in PC-DF-DH, $a=\alpha$. The complex transmittance of the sample (i.e., ${U_{mn}e^{i\theta_{mn}}}/{U_{mn}^{\mathrm{in}}}$) can be retrieved by the following calculation

${U_{mn}e^{i\theta_{mn}}}/{U_{mn}^{\mathrm{in}}}=\left( E_{mn}^{DF-DH}-E_{mn}^{DF-DH\_bg} \right)/{\sqrt{\alpha}E_{mn}^{DH\_bg}}$. (S21)

In the case of PC-DF-DH, an OPD image of the sample can be obtained by adding the OPD map loaded to the SLM to the complex transmittance map obtained by Eq. (S21).

In practice, different offset OPD is added to the interferometric term obtained in each measurement, such that the field $E$ in Eqs. (S18) – (S20) is replaced by $\tilde{E}$, where $\tilde{E}$ can be expressed as $\tilde{E}_{mn}^{DF-DH}=E_{mn}^{DF-DH}e^{-i\left( \theta_{0}+\theta_{1} \right)}$, $\tilde{E}_{mn}^{DF-DH\_bg}=E_{mn}^{DF-DH\_bg}e^{-i\theta_{0}}$ and $\tilde{E}_{mn}^{DH\_bg}=E_{mn}^{DH\_bg}e^{-i\left( \theta_{0}+\theta_{2} \right)}$ [note that $\theta_{0}$, $\theta_{1}$ and $\theta_{2}$ are independent of the spatial coordinate $\left( m,n \right)$]. Therefore, the OPD image can be obtained by

$\theta_{mn}=\angle\left[ \left( \tilde{E}_{mn}^{DF-DH}e^{i\theta_{1}}-\tilde{E}_{mn}^{DF-DH\_bg} \right)/{\sqrt{\alpha}E_{mn}^{DH\_bg}e^{i\theta_{2}}} \right]$. (S22)

To determine the offset OPD differences (i.e., $\theta_{1}$ and $\theta_{2}$) in Eq. (S22), a virtual OPD object is loaded on the SLM in the empty area of the FOV where the sample of interest does not exist when measuring $\tilde{E}_{mn}^{DF-DH}$ (see “measurement result” box in Fig. S3). $\theta_{1}$ can be determined such that the spatial OPD standard deviation of $\theta_{mn}$ reconstructed by Eq. (S22) in the empty area is minimized (see the region indicated by the red rectangle in the centre panel in the “reconstruction” box in Fig. S3). $\theta_{2}$ can be determined such that $\theta_{mn}$ of the virtual OPD object shows good agreement with the OPD values measured by DH beforehand (see the right panel in the “reconstruction” box in Fig. S3). Note that the virtual OPD object is made not to become the maximum OPD object in the FOV so that it does not limit the rate by which amount of the light illuminating the sample can be increased in PC-DF-DH. The temporal OPD change between two frames, $\theta_{mn} \left( t_{2} \right)-\theta_{mn} \left( t_{1} \right)$, can also be calculated by

$\theta_{mn} \left( t_{2} \right)-\theta_{mn} \left( t_{1} \right)=\angle\left[ \left\{ \tilde{E}_{mn}^{DF-DH} \left( t_{2} \right)e^{i\theta_{3}}-\tilde{E}_{mn}^{DF-DH} \left( t_{1} \right) \right\}/{\sqrt{\alpha}E_{mn}^{DH\_bg}e^{i\theta_{2}}} \right]$ (S23)

where $t_{1}$ and $t_{2}$ represent two different time, while $\theta_{3}$ shows the offset OPD difference between the two time frames. In Eq. (S23), $\tilde{E}_{mn}^{DF-DH\_bg}$ and $\theta_{1}$ in Eq. (S22) is replaced by $\tilde{E}_{mn}^{DF-DH} \left( t_{1} \right)$ and $\theta_{3}$, respectively. $\theta_{3}$ can be determined such that the spatial OPD standard deviation of $\theta_{mn} \left( t_{2} \right)-\theta_{mn} \left( t_{1} \right)$reconstructed by Eq. (S23) in the empty area is minimized.

**
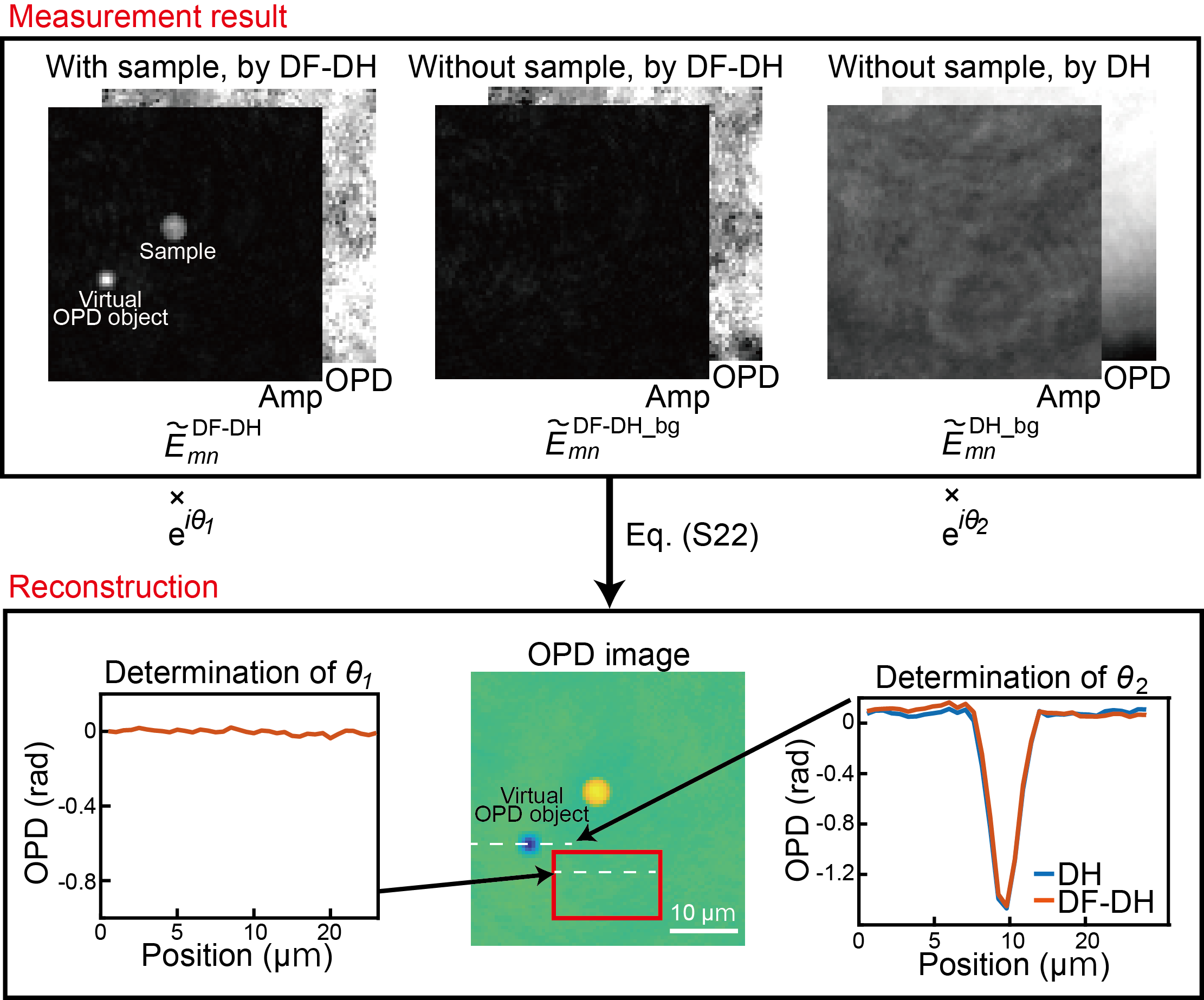
**

**Fig. S3 | Determination of the offset OPD differences required for OPD reconstruction with DF-DH.** The “measurement result” box shows the complex amplitude of the interferometric term obtained in each measurement (left: $\tilde{E}_{mn}^{DF-DH}$ measured by DF-DH with the sample, centre: $\tilde{E}_{mn}^{DF-DH\_bg}$ by DF-DH without the sample, right: $\tilde{E}_{mn}^{DH\_bg}$ by DH without the sample). To determine $\theta_{1}$ and $\theta_{2}$, a virtual OPD object is loaded on the SLM in the empty area of the FOV where the sample of interest does not exist when measuring $\tilde{E}_{mn}^{DF-DH}$ (see the left panel in the “measurement result” box). $\theta_{1}$ can be determined such that the spatial OPD standard deviation of $\theta_{mn}$ reconstructed by Eq. (S22) at the empty area (the region indicated by the red rectangle in the centre panel in the “reconstruction” box) is minimized. The left panel in the “reconstruction” box shows the OPD cross-section along the white dashed line in the region indicated by the red rectangle. $\theta_{2}$ can be determined such that the OPD values of the virtual OPD object (orange profile in the right panel in the “reconstruction” box) shows good agreement with the OPD values measured by DH beforehand (blue profile in the right panel in the “reconstruction” box).

1. **Effect of the DF mask when imaging low-spatial-frequency structures**

In practice, the DF mask has a finite size and therefore causes the high-pass filtering effect. Mathematically, this means that $U_{00}$ in Eqs. (S18) and (S19) contains some low-spatial-frequency amplitude and OPD distributions. The result is the Halo-like artifact appearing in the small-OPD regime of the dynamic range due to the loss of the low-spatial-frequency information. For example, Fig. S4 compares the photothermal images of water’s MIR absorption measured by DH and ADRIF-DH. The photothermal contrast that represents the MIR spot area is relatively large (~30 μm × 70 μm) in FOV, representing the situation where the low-spatial-frequency information is dominant. In such a case, a Halo-like artifact can be found in the result obtained by ADRIFT-DH. This problem could be mitigated by using a smaller DF mask.


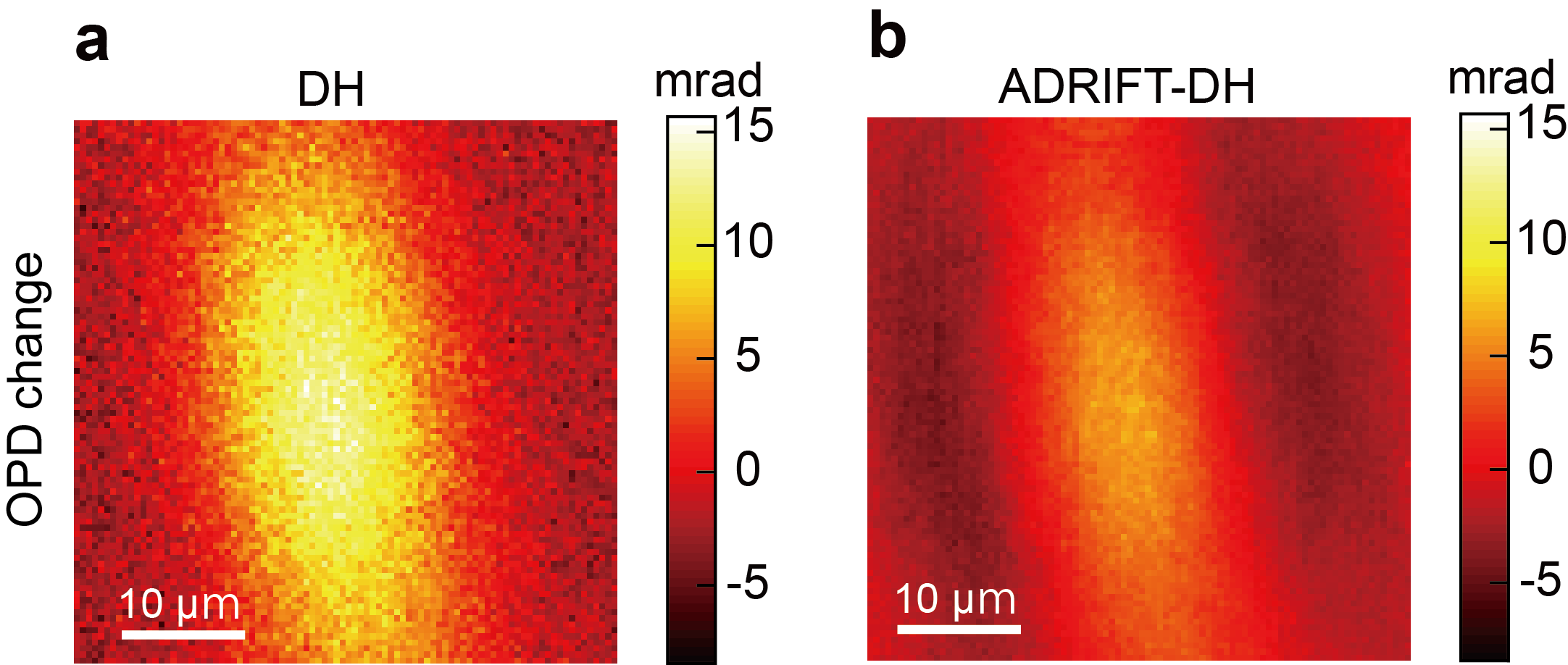


**Fig. S4 | Comparison of MIR photothermal phase images measured by DH and ADRIFT-DH when observing a non-sparse MIR absorber.** OPD change due to absorption of the MIR pump light by water measured by **a,** DH and **b,** ADRIFT-DH. The MIR pump laser is tuned to 1,550 cm^-1^. The photothermal contrast in DH reflects the beam profile of the MIR pump light.

One needs to be careful, however, that the loss of low-spatial-frequency information occurs to only the smaller-OPD regime of the dynamic range, but not necessarily to the larger-OPD regime. This is because the OPD map loaded to the SLM used for phase cancellation is calculated based on the measurement result obtained by DH where the DF mask is not inserted. This is why the OPD image of the cell obtained by ADRIFT-DH at the MIR-OFF state shows the same image without distortion as that obtained by DH (i.e., Fig. 5a in the main text).

Furthermore, we note that, in most cases, the low-spatial-frequency information would not become dominant in the OPD distribution after phase cancellation. First, this is because continuous OPD structures created by a natural sample such as a cell is cancelled by the discrete OPD structures created by the SLM. Therefore, the OPD structures after phase cancellation tend to dominantly contain high-spatial-frequency information. To confirm it, we perform numerical simulation to evaluate OPD distortion in ADRIFT-DH caused by the DF mask having a 50-μm diameter, which is the same as that used in our experiment (Fig. S5). The simulated OPD distribution obtained by PC-DF-DH shows good agreement with the true OPD distribution after phase cancellation where the high-spatial-frequency structures are dominant (see Fig. S5d). Second, when measuring small-OPD signals from scattering by nanoscale objects, we can expect the scattered images do not contain low-spatial-frequency information. In other word, we expect that the photothermal phase imaging, which is the demonstration of this work, is a special case where the low-spatial-frequency information becomes dominant after phase cancellation.


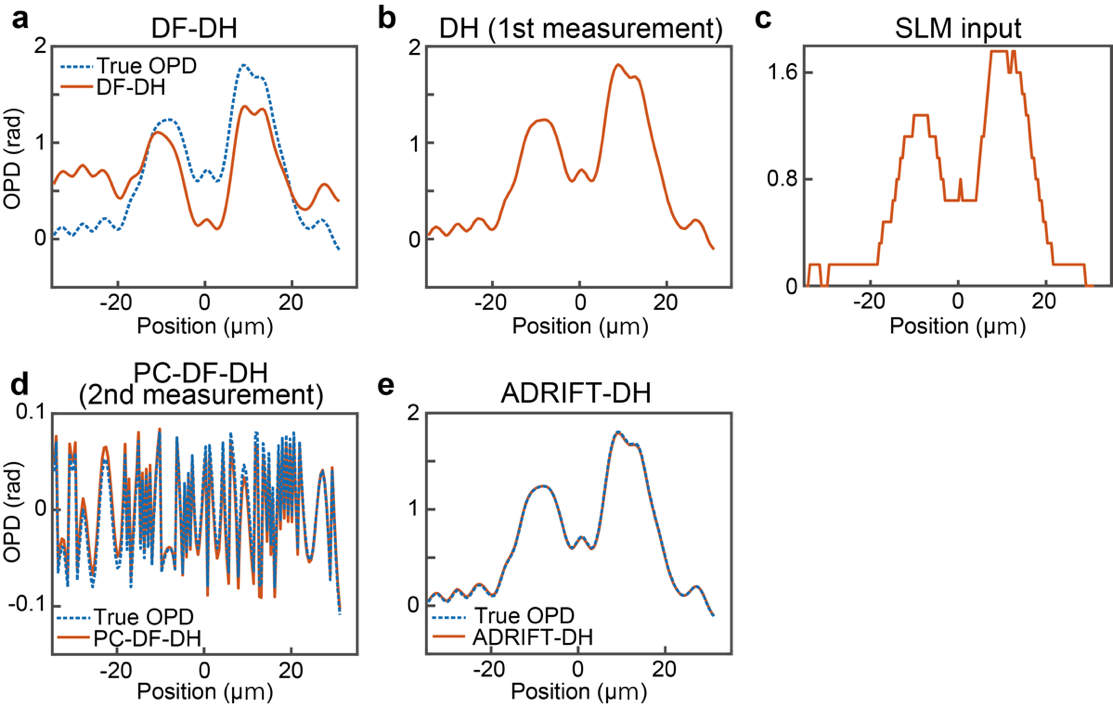


**Fig. S5 | Simulation result of the OPD distortion by DF-DH and ADRIFT-DH. a,** OPD distribution measured by DF-DH. The blue dotted curve shows a true OPD distribution and the orange curve shows the OPD distribution measured by DF-DH where the low spatial frequency component cannot be obtained due to the DF filtering. To mimic the actual experimental situation, the FOV is set to 75 μm (150 pixels) with the pixel size of 500 nm. To obtain the orange curve, the true OPD curve (blue) is first Fourier-transformed, and then the second lowest spatial-frequency components (i.e., the two pixels next to the DC pixel) in the Fourier domain is removed in DF-DH, which corresponds to high-pass filtering with a ~50 μm DF mask. The high-pass filtered frequency spectrum is finally inverse Fourier transformed to produce the orange curve. **b,** OPD distribution measured by DH (the first measurement in ADRIFT-DH). **c,** SLM input OPD map where the OPD distribution measured by DH is digitized. **d,** OPD distribution after phase cancellation. The blue dotted curve shows the true OPD distribution obtained by subtracting **c** from **b**. The orange curve shows the OPD distribution measured by PC-DF-DH (the second measurement in ADRIFT-DH) obtained by the same numerical high-pass filtering procedure as that described in **a**, where the high-spatial-frequency structures are dominant. **e,** Reconstruction of the OPD distribution obtained by ADRIFT-DH. The blue dotted curve shows the true OPD distribution and the orange curve shows the reconstruction by ADRIFT-DH (i.e., addition of the orange-curve OPD distributions shown in **c** and **d**).

**Reference**

1. Chen, S. *et al*. Phase sensitivity of off-axis digital holography. *Optics Letters.* **43**, 4993-4996 (2018).
